# Bronchial stenosis secondary to systemic paracoccidioidomycosis

**DOI:** 10.1590/0037-8682-0343-2023

**Published:** 2023-09-22

**Authors:** Daren Esteban Araque Gualtero, Diego Augusto Moreno Diaz, Javier Enrique Fajardo Rivero, Julio Cesar Mantilla, Stefano Valsangiacomo

**Affiliations:** 1 Facultad de Medicina de la Universidad Industrial de Santander, Bucaramanga, Santander, Colombia.

A 65-year-old man presented with dyspnea during moderate physical exertion, a persistent nonproductive cough, and progressive clinical deterioration. Upon examination, a perforated nasal septum was observed, along with decreased breath sounds on the right side and expiratory stridor. Paraclinical tests revealed a high neutrophil count in the blood, and contrast-enhanced chest tomography showed total stenosis of the right main bronchus (Figure 1). Further investigations, including spirometry, bronchoscopy, and nasal mucosa biopsy, confirmed the presence of *Paracoccidioides* spp. (Figure 2 and 3). The patient was treated with amphotericin B deoxycholate, followed by a 6-month course of itraconazole. Follow-up assessments indicated satisfactory progress, with improved symptoms and no need for supplemental oxygen at the time of hospital discharge.

**FIGURE 1:**
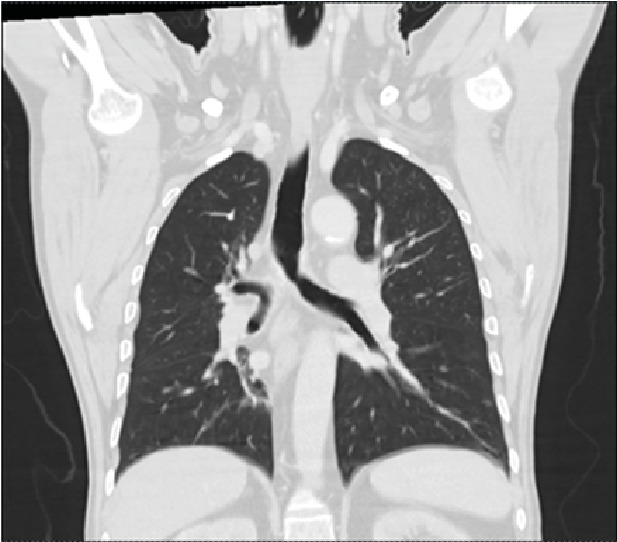
Chest tomography. Evidence of total stenosis of the right source bronchus.

**FIGURE 2:**
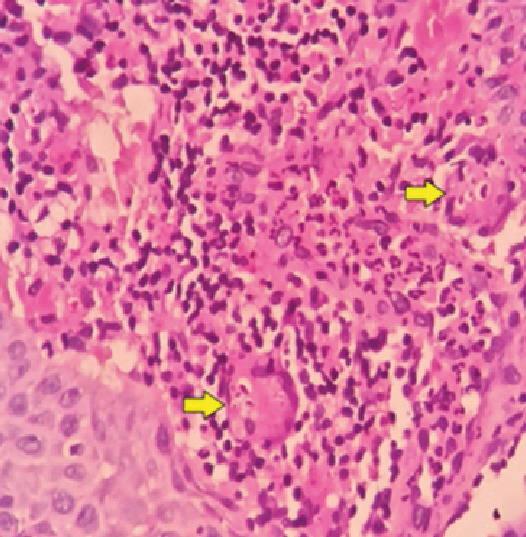
Microphotograph of mucosa of the nasal septum (400×). Granulomas with multinucleated giant cells and multigerm yeast inside (arrows).

**FIGURE 3:**
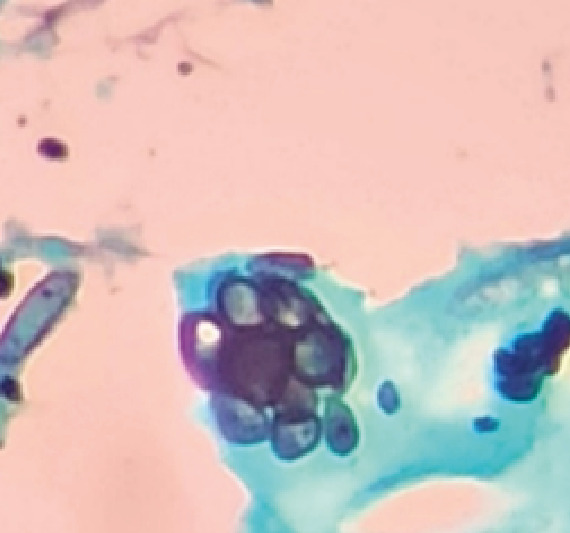
Morphological details of *Paracoccidioides* spp. revealing a distinctive "ship's wheel" morphology, as observed using Grocott's methenamine silver stain.

Paracoccidioidomycosis, a systemic fungal infection caused by *Paracoccidioides* spp., is primarily contracted through the inhalation of spores^1^. The infection can manifest as an acute/subacute form affecting 5-25% of those infected, or as a chronic form, which manifests gradually with symptoms such as cough, dyspnea, and physical manifestations including skin and oral lesions^2^. Notably, right source bronchus stenosis as a manifestation of Paracoccidioidomycosis is exceedingly rare. Although Paracoccidioidomycosis is more prevalent in South America, particularly in Colombia and Brazil, it has been reported globally, albeit in limited cases^3^. Recognizing this systemic manifestation is crucial in the differential diagnosis of respiratory conditions or granulomatous diseases involving the airways.

## ETHICAL CONSIDERATIONS

The study was approved by the Hospital Universitario de Santander Ethics Committee.

## References

[1] Queiroz FV, Peçanha PM, Rosa M, Baptista RM (2020). New insights on pulmonary paracoccidioidomycosis. Semin Respir Crit Care Med.

[2] Bocca AL, Amaral AC, Teixeira MM, Sato PK, Shikanai MA, Soares MS (2013). Paracoccidioidomycosis: eco-epidemiology, taxonomy and clinical and therapeutic issues. Future Microbiol.

[3] Peçanha PM, Peçanha PM, Grão TR, Rosa M, Falqueto A, Gonçalves SS (2022). Paracoccidioidomycosis: What we know and what is new in epidemiology, diagnosis, and treatment. J Fungi.

